# Social cognition and major depressive disorder: impact on psychosocial function and therapeutic opportunities

**DOI:** 10.1192/bjo.2021.794

**Published:** 2021-06-18

**Authors:** Michael Weightman, Bernhard Baune

**Affiliations:** 1 University of Adelaide; 2 University of Melbourne

## Abstract

**Aims:**

This poster aims to examine the impact of social cognitive deficits on psychosocial functioning in depressed patients, as well as summarise the utility of various evidence-based therapeutic interventions employed to target these deficits. The stated hypotheses were twofold: (1) that social cognitive impairment in major depressive disorder will correlate with poorer psychosocial functioning; and (2) that these deficits will respond to existing anti-depressant therapies.

**Background:**

Social cognition is an important adaptive trait that incorporates the identification, perception and interpretation of socially relevant information from the external world. It is frequently affected in major depressive disorder such that depressed patien

**Method:**

A review of the existing literature was performed in order to test the stated hypotheses. Pertinent sources were identified via the MEDLINE, EMBASE, PsycINFO, PubMed, Scopus and Google Scholar databases. A total of 107 studies met inclusion criteria for review.

**Result:**

Impaired social cognitive performance in depressed patients correlated with poorer psychosocial functioning across the key domains of general cognitive functioning and quality of life. Many current anti-depressant therapies were found to have a normalising effect on the social cognitive abilities of depressed subjects, both at a neural and functional level. Anti-depressant medications, in particular citalopram and reboxetine, appeared to correct facial affect recognition deficits, while a psychotherapeutic approach demonstrated improvements in theory of mind and negative interpretive bias. Data relating to other common treatments, such as electroconvulsive therapy, are limited.

**Conclusion:**

The impact and treatment of social cognitive deficits in major depressive disorder is an important emerging field. The social cognitive deficits evident in depressed patients are sometimes subtle, but afford a significant functional impact. Additionally, it appears these impairments are at least partially reversible using anti-depressants or psychotherapy.

